# Supplementary material for: Dissecting the puzzle of tectonic lid regimes in terrestrial planets
Source: Nat Commun. 2025 Nov 24;16:10037. doi: 10.1038/s41467-025-65943-1 (PMC12644805; doi:10.1038/s41467-025-65943-1)
Supplement: Supplementary file 1 — supplementary Information file [file 41467_2025_65943_MOESM1_ESM.pdf]

Supplementary Information for

Dissecting the puzzle of tectonic lid regimes in terrestrial planets

Tianyang Lyu[1], Maxim D. Ballmer[2,*], Zhong-Hai Li[3,*], Man Hoi Lee[1,4,*], Jun Yan[5], Benjun Wu[6], Guochun Zhao[1,7]

[1]NWU-HKU Joint Center of Earth and Planetary Sciences, Department of Earth and Planetary Sciences, The University of Hong Kong, Hong Kong, China

[2]Department of Earth Sciences, University College London, London, UK

[3]State Key Laboratory of Earth System Numerical Modeling and Application, College of Earth and Planetary Sciences, University of Chinese Academy of Sciences, Beijing, China

[4]Department of Physics, The University of Hong Kong, Hong Kong, China

[5]Department of Earth Sciences, Freie Universität Berlin, Berlin, Germany

[6]School of Earth Sciences and Engineering, Nanjing University, Nanjing, China

[7]State Key Laboratory of Continental Dynamics, Department of Geology, Northwest University, Xi'an, China

*** Corresponding authors:**
M. D. Ballmer <m.ballmer@ucl.ac.uk>, Z.-H. Li <li.zhonghai@ucas.ac.cn>,
and M. H. Lee <mhlee@hku.hk>

This PDF file includes:

Text S1-S4

Figures S1-S9

Table S1-S4

Supplementary Material references

Text S1. Effects of upper-mantle activation energy

The upper mantle is a key region for understanding the dynamics of the Earth's interior, as it influences the patterns of mantle convection and lithospheric deformation. Compared to deeper regions such as the lower mantle or core, the rheology of the upper mantle is experimentally relatively well-constrained[1]. However, the dominant deformation mechanism (diffusion vs. dislocation creep) depends on stress and water content[2]. In a simplified linear rheological description, we explore the upper-mantle activation energy, E_{UM}; high E_{UM} of 300~360 kJ/mol are appropriate for dominant diffusion creep, whereas relatively low E_{UM} of 100~200 kJ/mol can mimic the effects of dislocation creep on boundary-layer dynamics[3].

In this study, we also explore the effects of upper-mantle activation energy and surface yield stress (for a fixed thermal condition with Rh = 1.0×10^{-11} W/kg and T_{CMB} = 4000 K). Accordingly, eight model cases (i.e., with E_{UM} = 200 kJ/mol, Rh = 1.0×10^{-11} W/kg and T_{CMB} = 4000 K, but variable surface yield stress; i.e. the first eight cases in Supplementary Data 1) are shared between both series as a common baseline. Fig. S1a shows the average of plateness and surface mobility, and their standard deviations, for models with different E_{UM} and σ_s. As above, we use the fractions of time in different base regimes as diagnostics for tectonic regimes (Fig. S1b). For intermediate E_{UM}, the effect of increasing σ_s is to promote episodic-squishy, and then plutonic-squishy lid regimes (i.e., at the expense of plate tectonics). For high E_{UM}, the effect of increasing σ_s is instead to promote episodic, and then stagnant-lid behavior. When E_{UM} has a high

value, the cool lithosphere exhibits a higher viscosity at a given viscosity of the hot asthenosphere. Consequently, the stresses at the base of the lithosphere are too small to cause yielding. Meanwhile, the low-viscosity upper mantle (with high mantle temperatures, see Fig. S2b) leads to more accelerated mantle convection, further decoupling the upper mantle and lithosphere. The effect of high E_{UM} to promote episodic and stagnant-lid regimes is similar to that of high thermal conditions (Fig. 5a), for which decoupling of the lithosphere is promoted by a hot upper mantle.

For low E_{UM}, mobile-lid behavior is dominant over the whole range of σ_s explored here. Low E_{UM} decreases the viscosity of cold lithosphere, enhancing the coupling between the mantle and the lithosphere, and thus sustaining the initiation and maintenance of plate tectonics. Indeed, this enhanced coupling ensures that a mobile lid persists even when the viscosity contrast between the surface and the asthenosphere exceeds the canonical ~10^4 threshold generally required for stagnant-lid convection[4].

Notably, the parameter range of models that exhibit a plutonic-squishy lid is quite narrow, only occurring at high surface yield stress ($\sigma_s \gtrsim 210$ MPa) and moderate activation energy ($E_{UM} = 200$ kJ/mol) (Fig. S1b). This is because, for plutonic-squishy lid behavior, the middle layers of the lithosphere need to be sufficiently soft (otherwise, coherent subduction would occur instead of dripping), while the top of the lithosphere must remain rigid. For low σ_s, the lithosphere yields at low stresses, such that a mobile lid emerges. Similarly, for low E_{UM}, the lithosphere is thin and prone to wholesale yielding (plate subduction; mobile lid). In turn, for high E_{UM}, the base and core of the

lithosphere are too rigid, such that a stagnant lid emerges. As shown in many previous works, the stagnant lid also tends to emerge for high surface yield stresses.

A similar balance is required to stabilize the episodic-squishy and plutonic-squishy lid regimes as a function of mantle thermal condition (Fig. 5). These regimes only occur at intermediate thermal conditions, for which sufficient plutonism occurs to weaken the mid-lithosphere, but mantle-plate coupling is still sufficiently strong to permit localized deformation.

Text S2. Resolution test

To validate our main conclusions, we repeated selected cases with an increased resolution. The high-resolution cases are performed in a 2D quarter-annulus geometry with 192 x 96 cells. This corresponds to a grid resolution that is 1.5x higher than for the cases presented in the main text. Radial grid refinement is applied, increasing resolution by up to a factor of two near the surface and the core–mantle boundary (CMB), resulting in a radial grid spacing of approximately 18 km at the surface and 27 km at the CMB. Since our main purpose in comparing cases of different resolutions is to determine if they achieve the same tectonic regime in the statistical steady-state, we selected the last two billion years of model time (i.e., from 6 to 8 Gyr) for this comparison. Except for the number of grid cells and selected model time, the parameters for the high-resolution models are identical to their corresponding low-resolution cases, including the average number of tracers per cell. Following Lourenço

et al.[5], we place the intrusions at mid-lithospheric depths, as this promotes the plutonic-squishy-lid regime, especially at high resolution.

Our analysis of high-resolution cases (Fig. S4 and Table S3) reveals that the overall distribution of cases across the tectonic regime diagram is very similar to that of low-resolution cases. We provide a brief description of the main features for each tectonic regime, confirming their similarity to the low-resolution models:

1. Mobile Lid regime: High-resolution models with low surface yield stress display high plateness and mobility, indicating efficient plate subduction and persistent lid fragmentation, consistent with low-resolution cases.

2. Stagnant Lid regime: At high surface yield stress and high thermal conditions, high-resolution models display minimal lithospheric displacement and deformation, with low surface mobility and velocity, similar to corresponding low-resolution cases.

3. Episodic Lid regime: Episodic-lid cases with alternating mobile and stagnant episodes occur as a transitional regime between the mobile and stagnant lid cases (i.e., at intermediate surface yield stresses and high thermal conditions).

4. Episodic-Squishy Lid regime: High-resolution models with intermediate surface yield stresses and thermal conditions exhibit alternating phases of mobile-lid and plutonic-squishy lid behavior, similar to the low-resolution cases.

5. At high surface yield stresses and intermediate thermal conditions (yellow star in Fig. S4b), high-resolution models also show the typical characteristics of the episodic-squishy regime (ES). This prediction is in contrast to low-resolution cases, which

display “pure” plutonic-squishy lid (PS) behavior at these conditions. We note that the ES and PS regimes are indistinguishable except for intermittent phases of mobile-lid activity in ES. In the particular case discussed (yellow star in Fig. S4), these mobile phases are notably short (global-scale resurfacing events).

6. Sluggish Lid regime: At low thermal conditions, plateness remains high while mobility is lower, reflecting passive lithosphere dragging instead of active subduction, consistent with low-resolution cases.

Although we have not re-run all our models at high resolution, the similar distribution of the tectonic regimes of high- and low-resolution cases (Figs. S4 vs. 3) corroborates the validity of low-resolution cases presented in the main text. Almost all high-resolution cases explored here display the same tectonic behavior as in the corresponding low-resolution case; the characteristic statistics of mobility and plateness (Table S3) as well as overall dynamics are also analogous to low-resolution models. Only the case with a surface yield stress coefficient of 300 MPa and intermediate thermal conditions predicts short global-scale overturns in addition to continuous plutonic-squishy lid behavior instead of “pure” plutonic-squishy lid behavior (i.e., ES instead of PS behavior). This discrepancy corroborates one of our main findings: the pure plutonic-squishy lid regime occurs over a rather narrow parameter space in our statistical steady-state models.

Text S3. Initial potential temperature test

We also explored the effects of initial condition, by running additional test cases with initial potential temperatures (T_{p_init}) of 1700 K and 2100 K. We ran ten test cases with T_{p_init} = 1700 K, and another ten test cases with T_{p_init} = 2100 K (see Fig. S5). As usual, we focus on the statistical steady-state, analyzing the last two billion years of model time (i.e., from 6 to 8 Gyr). Our results show that in all cases, regardless of the initial geotherm, the predicted tectonic regime remains the same (see Fig. S5 and Table S4). Therefore, we conclude that, unlike previous studies[6,7], the initial thermal structure does not strongly affect the preferred tectonic mechanism of the planet, at least in the statistical steady-state. We did not scrutinize how the transient evolution of the planet depends in detail on the initial condition, but we find that average mantle temperatures (and thus viscosity profiles) converge after 1-2 Gyr for cases with different T_{p_init}.

These findings support the robustness of our conclusions and expand our understanding of the factors that control the development of different tectonic regimes. The robustness of our results is likely due to the regulating effect of mantle melting[8] on mantle temperatures, and thus on the dynamics of the plate-mantle system.

Text S4. Transient behavior in tectonic regime transitions

To investigate the role of transient behavior in tectonic regime transitions, we conducted a series of restart experiments. In these restart experiments, simulations are re-initialized from well-established statistical steady states of four regimes: mobile lid

(ML), stagnant lid (ST), episodic-squishy lid (ESL), and sluggish lid (SL) at 10 Gyr model time (Fig. S7). In each restart experiment, only one critical parameter—surface yield stress or upper-mantle activation energy—is modified upon restart. These additional test cases provide insight into the application of our suite of steady-state cases (see main text and Suppl. Text S1-S2) to the transient evolution of terrestrial planets.

Comparison of these "restart experiments" to the results of the corresponding steady-state models can address two key issues: First, by comparison to the steady-state model, from which they are restarted, they address what would happen during a transient stage of planetary evolution (e.g., during a stage in which the effective surface yield stress abruptly changes) (see Fig. S7). Second, by comparison to the steady-state case with the same parameters, they also allow to study the effects of initial conditions, and thereby, the relevance of hysteresis (note that the steady-state case has been started from a radially-symmetric geotherm with boundary layers; the restart experiment from a 2D thermochemical field predicted by a steady-state case with different parameters after 10 Gyr model time).

We focus on investigating restart experiments with modified surface yield stress (σ_s) and upper-mantle activation energy (E_{UM}), because the thermal conditions are expected to change very slowly during planetary evolution. Specifically, we focus on five pairs of restart experiments (Fig. S8): (a-b) Rh200cc300 ↔ Rh200cc40; (c-d) Rh125cc300 ↔ Rh125cc160; (e-f) Rh100cc160 ↔ Rh100cc80; (g-h) Rh50cc300 ↔

Rh50cc80; and (i-j) Ea250cc300 ↔Ea150cc300.

As shown in Fig. S8a, the restart experiments corresponding to Figure S7a,c,e,g involve decreasing σ_s, and therefore, regime transitions from the right to the left in the regime diagram, typically with increasing mobility, are expected. Figure S7a,c,e,g demonstrates that the regime transitions indeed occur as expected according to the regime map (Figs. 5b, S8a), and importantly, they occur almost instantaneously.

In turn, the restart experiments corresponding to Figure S7b,d,f,h involve increasing σ_s, and therefore, regime transitions from the left to the right in the regime diagram, typically with decreasing mobility, are expected. Figure S7b,d,f,h demonstrates that these regime transitions eventually occur as expected according to the regime map (Figs. 5b, S8a), but the transitions takes significantly longer time than the cases above (~0.1 to ~1.5 Gyr), sometimes involving an intermittent regime for a short time.

We also tested one pair of restart experiments with changes in E_{UM} (Fig. S8b), as shown in Figure S7i-j. Our results indicate that the transition from high-E_{UM} to low-E_{UM} (i.e., corresponding to a reduction of the viscosity contrast between the lithosphere and asthenosphere) occurs more quickly than the reverse transition (low-E_{UM} to high-E_{UM}), supporting the conclusion that changes that lead to a weaker lithosphere involve very fast regime transitions. In turn, regime transitions from a more mobile to a less mobile regime due to parameter changes that lead to a stronger lithosphere may be delayed (Fig. S7, right column). This result is generally consistent with the self-

stabilizing effect of mobile regimes (such as plate tectonics) due to hysteresis[9,10].

In any case, our results indicate that the impact of hysteresis is relatively weak in our models. Even though regime transitions are delayed during some of our restart experiments, they are just delayed by 0.1~1.5 Gyr, and only when the strength of the lithosphere is increased during restart. This result contrasts with previous studies (e.g., refs. [10,11]), which found that the ultimate tectonic regime reached (i.e., after much more than 1.5 Gyrs) depends on the initial condition, e.g., whether the model is restarted from a fully-developed mobile-lid or stagnant-lid case. Our tests with variable initial temperatures (Fig. S5) provide further support for the limited influence of initial conditions. We attribute this limited hysteresis to our general model setup, which includes self-consistent mantle melting, as well as intrusive and extrusive melt emplacement in the lithosphere. The related magmatic heat flux regulates mantle temperature, and thus tectonic evolution (e.g., ref. [8]). Magmatism also gives rise to lithospheric heterogeneity, which facilitates the formation of tectonic boundaries. Thus, our work emphasizes the self-regulating effects of mantle melting on plate-mantle coupling, and thus on the long-term (tectonic) evolution of Earth-like planets.

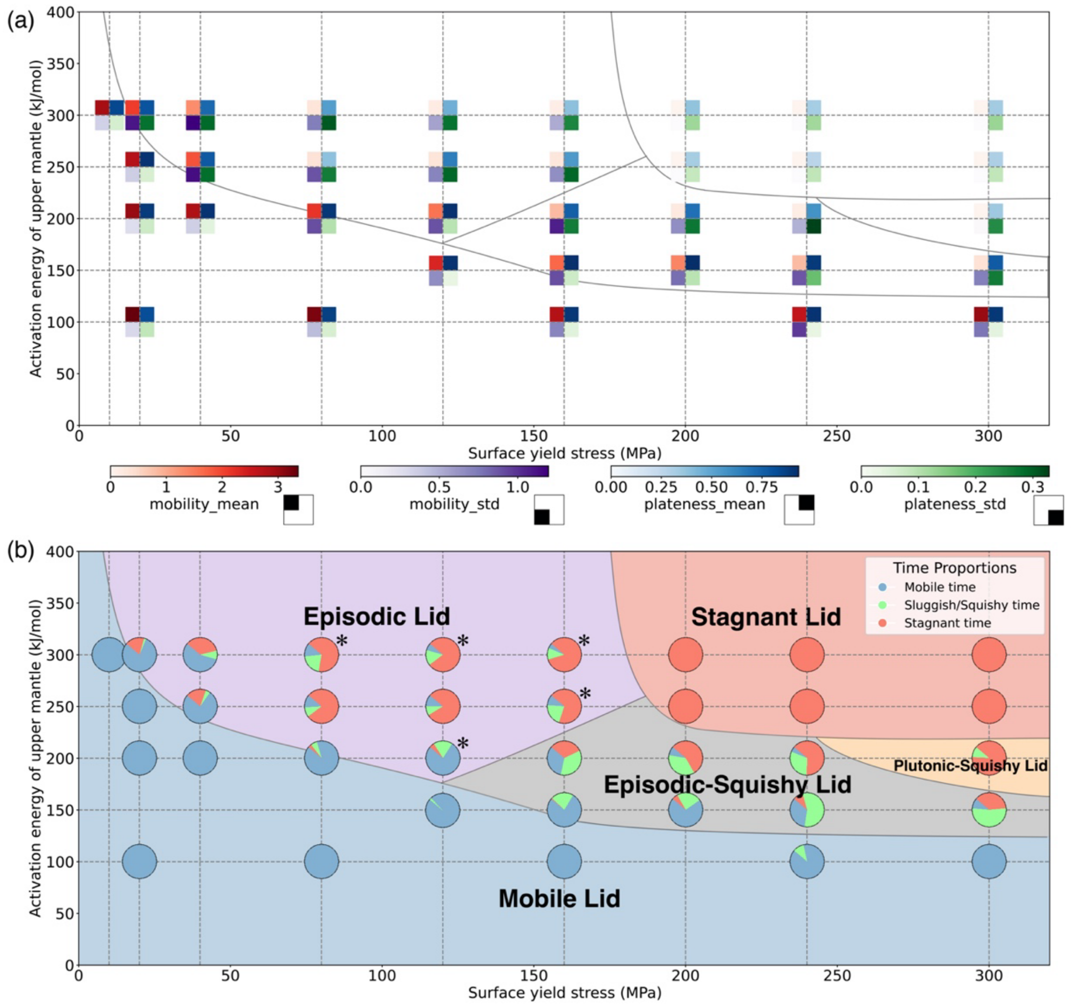

Fig. S1. (a) Representation of the time series for mobility (M) and plateness (P) (as in Fig. 2), summarized as its time-average (or mean) and variability (i.e., standard deviation) for all cases with variable surface yield stresses and upper-mantle activation energy. Within the same parameter space, panel (b) shows an overview of regimes (and their boundaries), as inferred from our quantitative criteria (see section Tectonic regimes). These criteria are used to quantify the model time durations of the three basic styles (i.e. mobile, sluggish/squishy, and stagnant) based on M and P (see scatterplot, each pie chart represents the relative contributions of each regime for the corresponding case). (*) incipient stagnant phases during the episodic lid overlap with the criteria of sluggish/squishy base regimes in terms of M and P (Table 1).

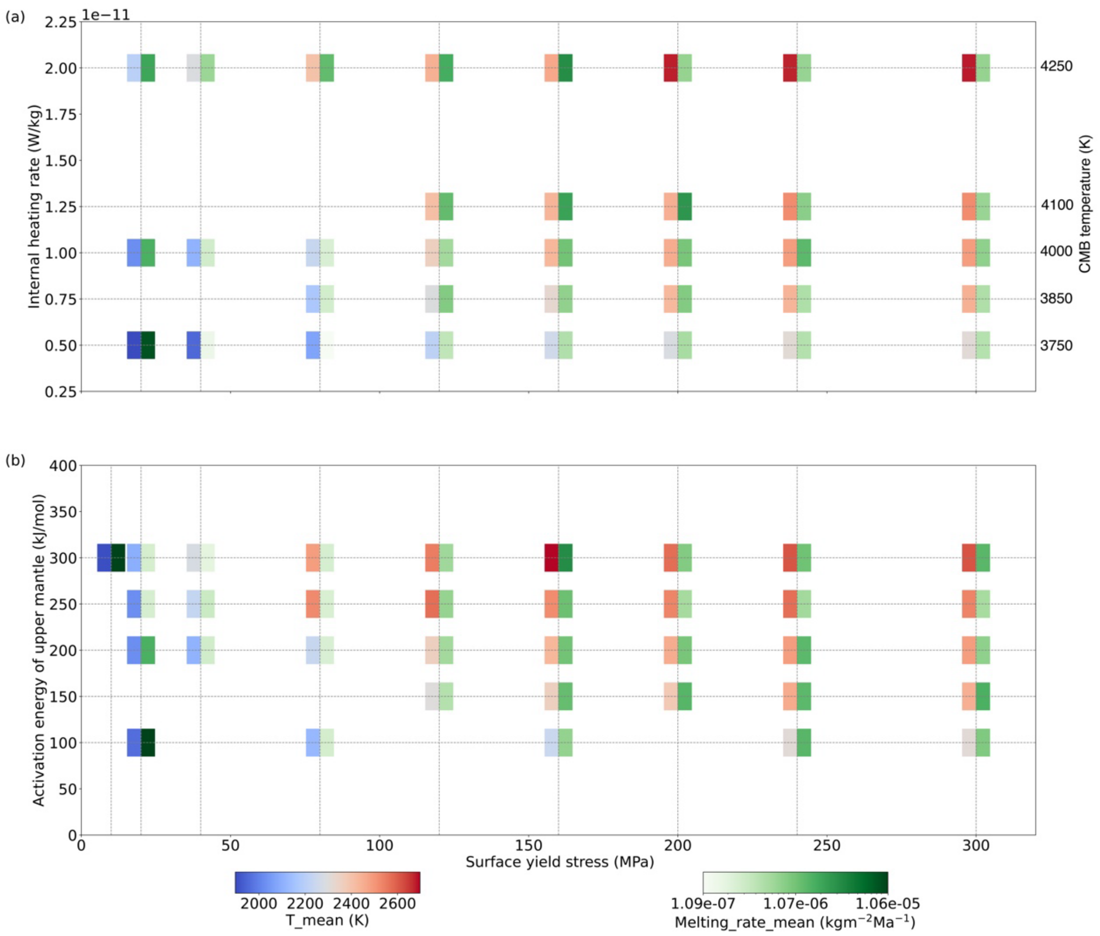

Fig. S2. Depiction of the time series for mantle temperature and melting rate, as illustrated in the example cases in Fig. 2, summarized as its time-average (or mean) for all cases. (a) Variable surface yield stress, internal heating rate and CMB temperature. (b) Variable surface yield stress and upper-mantle activation energy.

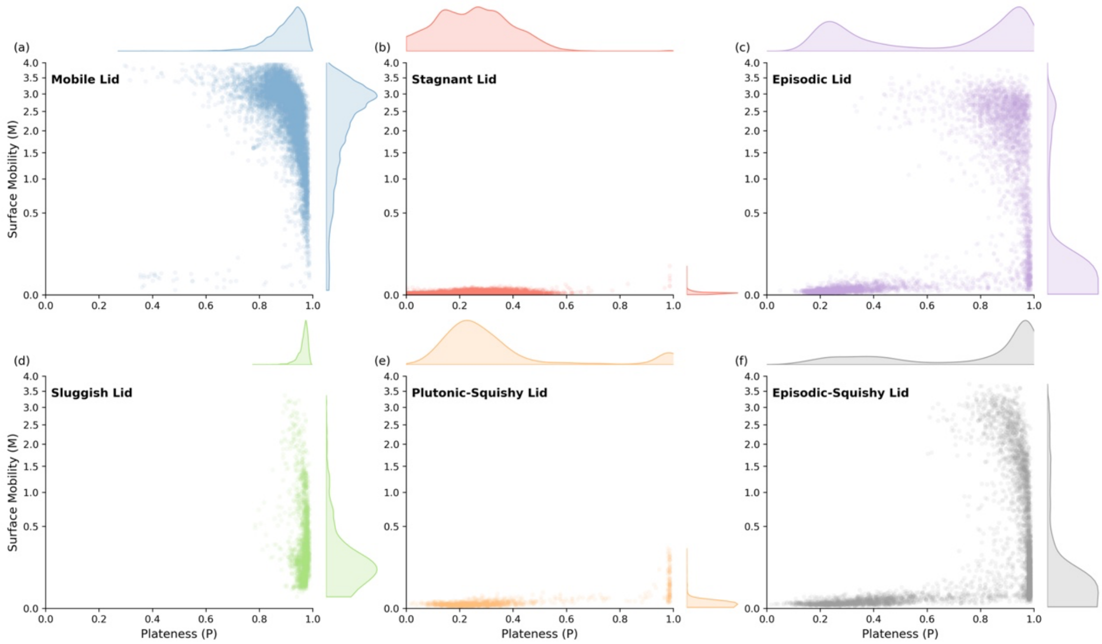

Fig. S3. Scatter plots illustrating the relationship between Plateness (P) and Surface Mobility (M) for various lid modes: (a) mobile lid, (b) stagnant lid, (c) episodic lid, (d) sluggish lid, (e) plutonic-squishy lid, and (f) episodic-squishy lid. The data points are derived from a unified collection of cases presented in Figs. 5 and S1, categorized according to their respective lid modes. The points are color-coded according to their respective lid mode and represent the entire range of points within the 6–10 Gyr time interval. Kernel density estimates (KDE) for P and M are shown at the top and right of each subplot, respectively, depicting the probability distributions of P and M.

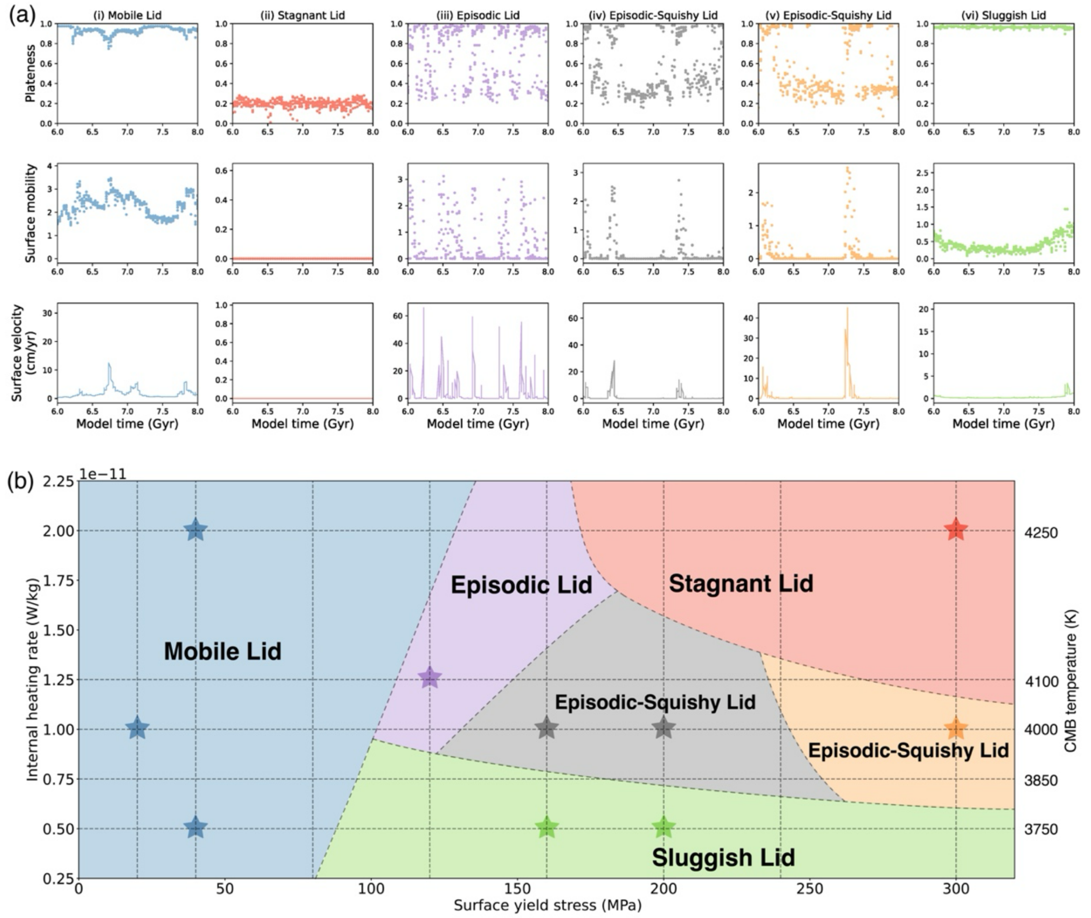

Fig. S4. Resolution test under high-resolution conditions (Table S3). (a) Plateness, mobility, and root-mean-square surface velocity over model time in the statistical steady state (the last 2 Gyr of a much longer model evolution) for six characteristic cases for each regime: (i) mobile lid: Rh50cc40_h, (ii) stagnant lid: Rh200cc300_h, (iii) episodic lid: Rh125cc120_h, (iv) episodic-squishy lid: Rh100cc160_h, (v) episodic-squishy lid: Rh100cc300_h, and (vi) sluggish lid: Rh50cc160_h. (b) Regime diagram of representative high-resolution cases selected from the parameter space of Fig. 5. The coordinates of the star-shaped symbols represent the selected parameters at each point, and the colors of those symbols correspond to the different lid types as in (a). For ease of comparison, the distribution of lid regimes (colored patches) in this figure is consistent with that in Fig. 5.

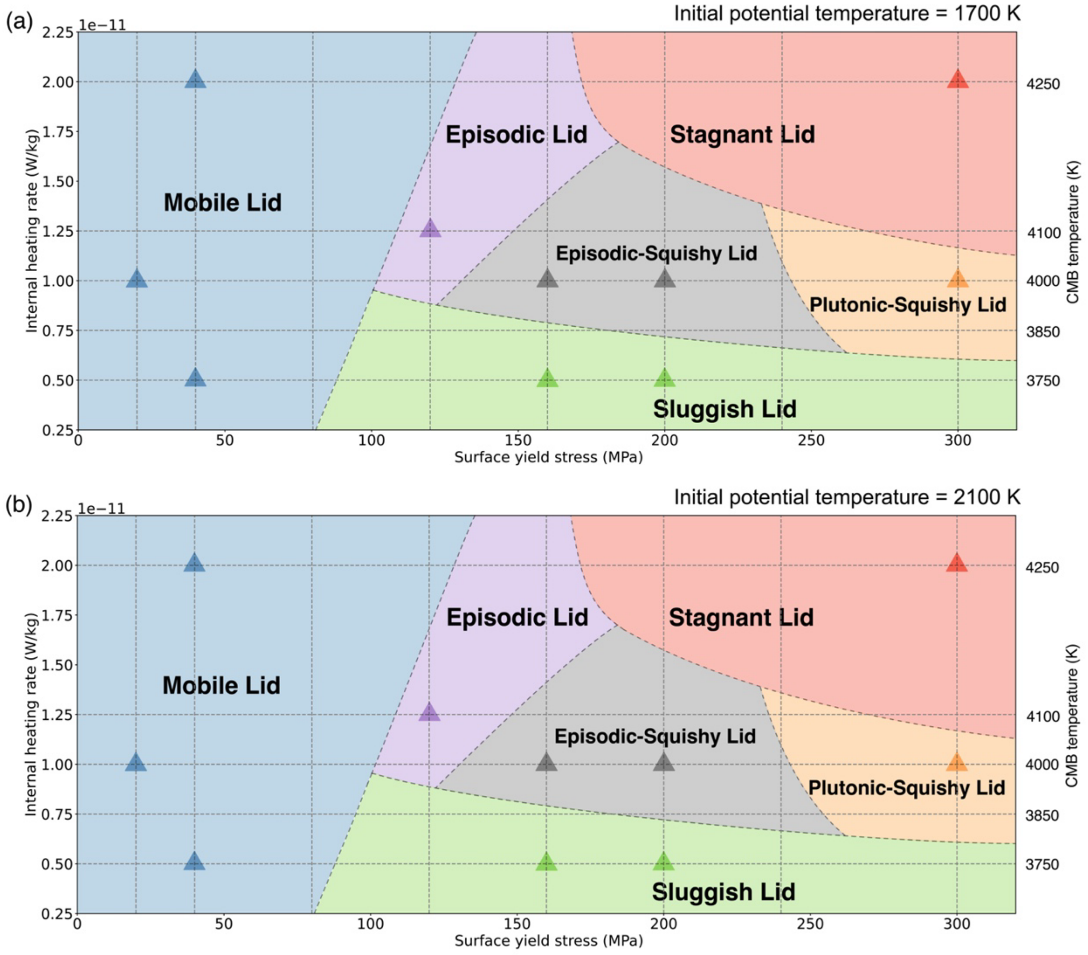

Fig. S5. Parameter test under different values of initial potential temperature (Table S4). (a) Regime diagram with an initial temperature of 1700 K, and other conditions identical to the cases selected in Fig. S4b. (b) Regime diagram with an initial temperature of 2100 K, and other conditions identical to the cases selected in Fig. S4b. The coordinates of the triangle-shaped symbols represent the selected parameters at each point, and the colors of those symbols correspond to the different lid types as in Fig. 2. For ease of comparison, the distribution of lid regimes (colored patches) in this figure is consistent with that in Fig. 5.

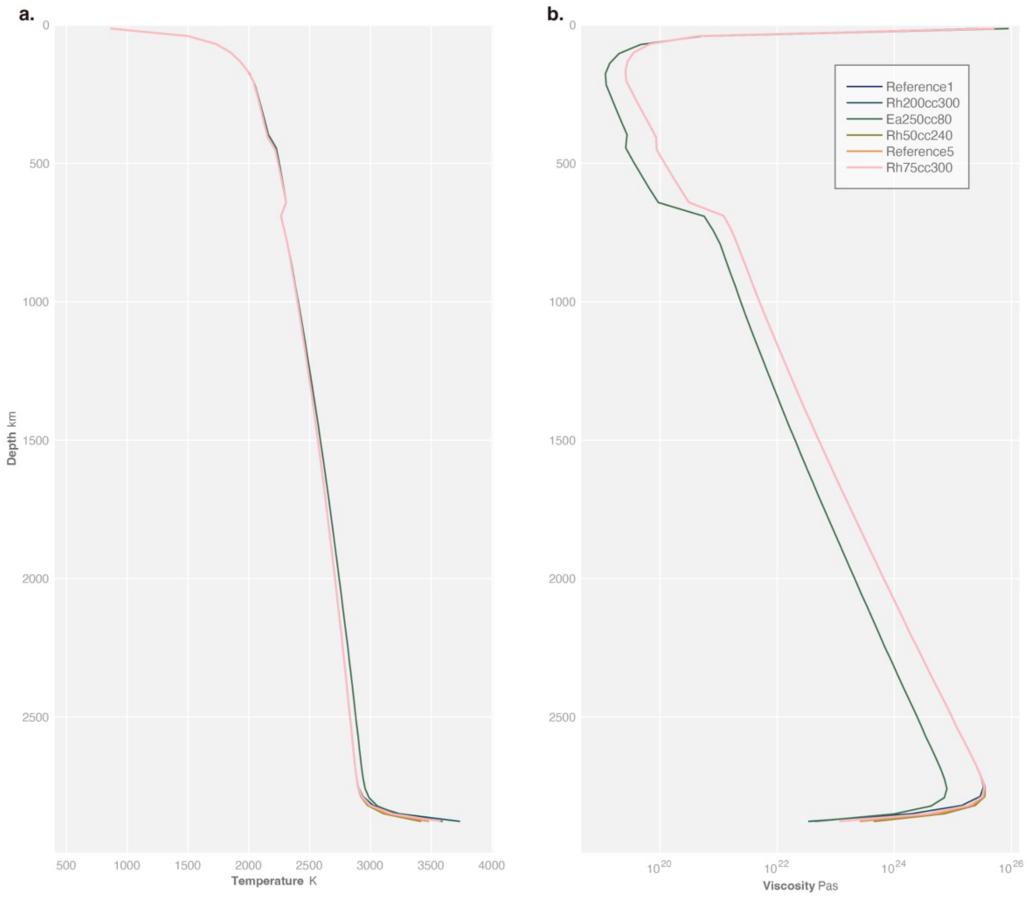

Fig. S6. Radial average profiles of (a) temperature and (b) viscosity for the same six cases as depicted in Fig. 1 at the initial condition.

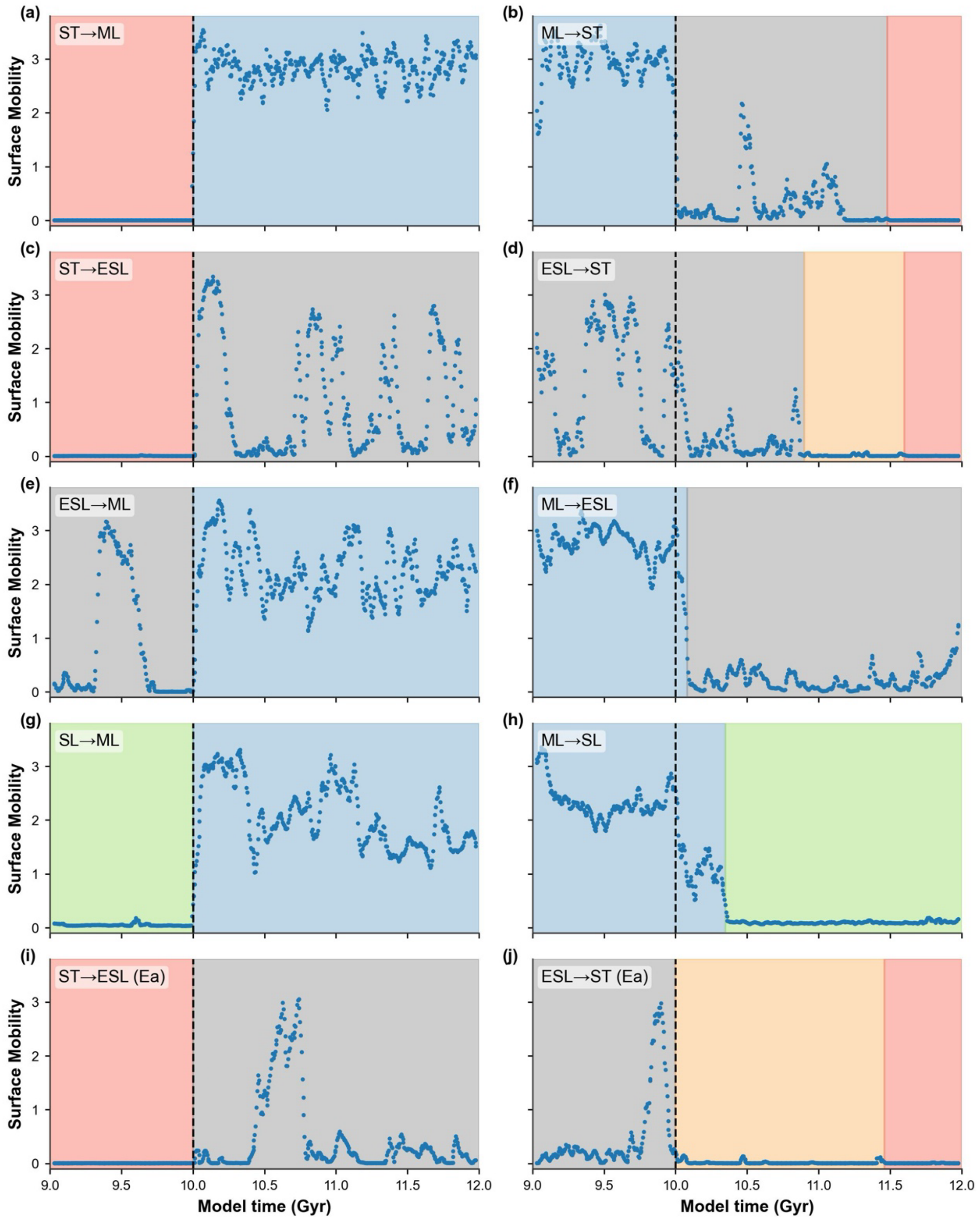

Fig. S7. Time evolution of surface mobility and tectonic regime transitions during restart experiments. Time series of surface mobility (M) over the model time of 9–12 Gyr for ten restart experiments. At 10 Gyr (vertical dashed line), each of these cases (a-j) is restarted from an "original" model result with different parameters. Accordingly, the mobility shown at <10 Gyr corresponds to that of the original case. At 10 Gyr, either the upper-mantle activation energy (panels marked by "Ea") or the surface yield stress are instantaneously changed (according to Fig. S8). Colored shading denotes the tectonic regime diagnosed at each timestep, following the lid mode classification and color scheme used in Fig. 2. White panel titles "X → Y" indicate the initial (X) and target (Y) regimes according to Fig. S8.

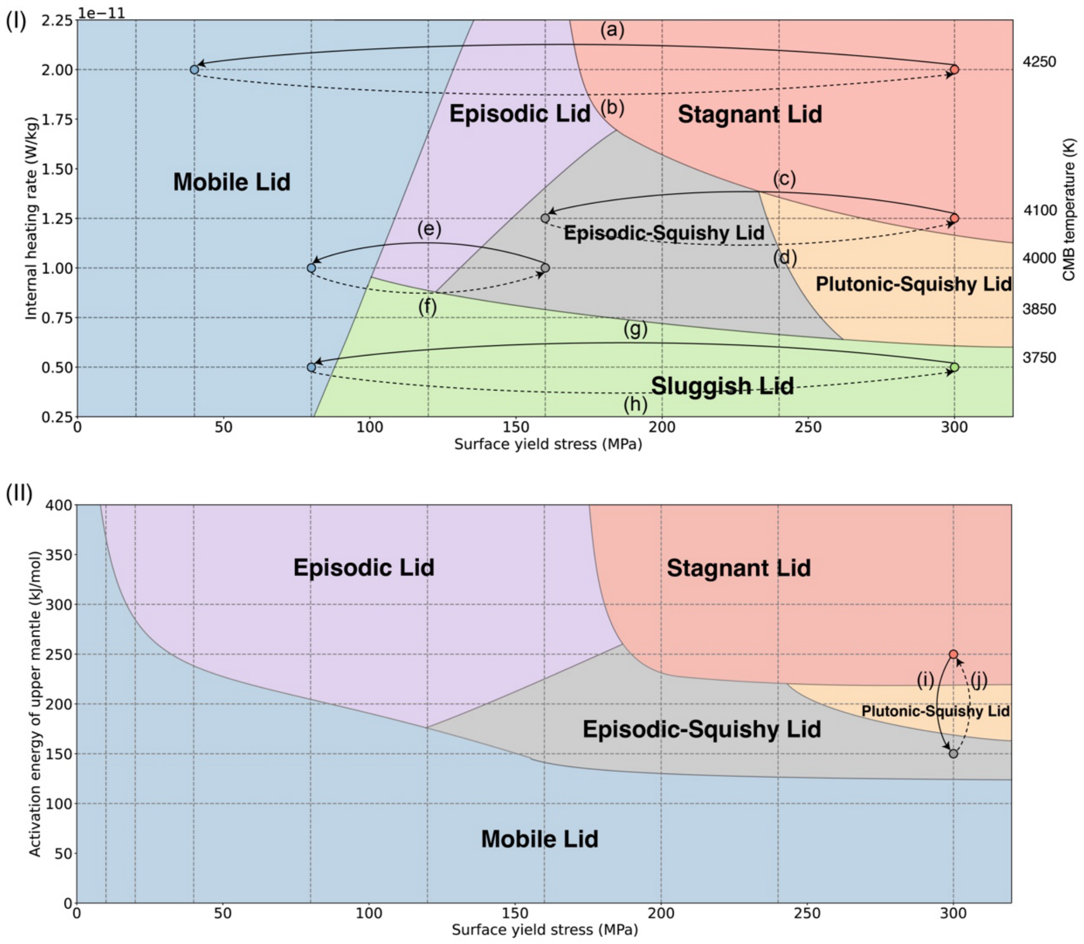

Fig. S8. Schematic lid-regime diagrams with parameter variations that govern restart-experiments in Fig. S7. Panels (I) and (II) present two distinct cross-sections through our parameter space. They correspond to the regime maps in (I) Fig. 5b and (II) Fig. S1b. At 10 Gyr model time (vertical dashed line in Fig. S7), each of the ten restart experiments (a–j as labelled) is run from the temperature and compositional fields of the last timestep of the case that is marked by the beginning of the arrow (i.e., as initial conditions of the restart experiment), but with instantaneously modified parameters (tip of each arrow).

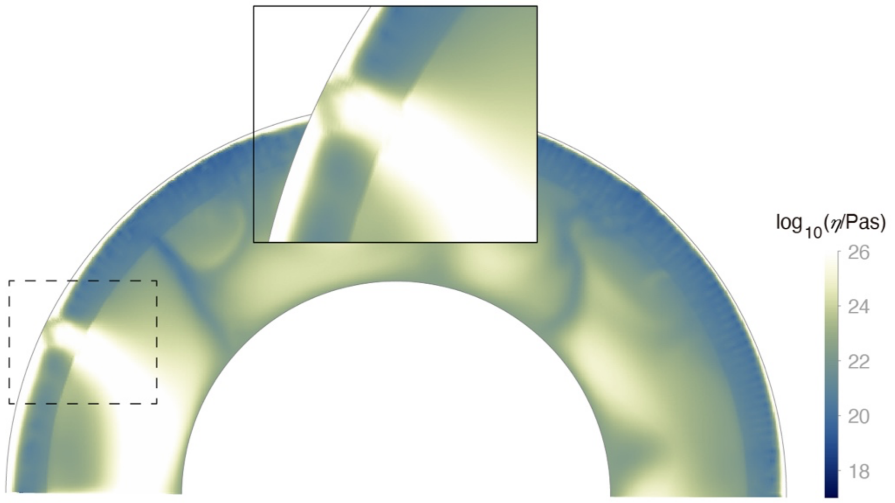

Fig. S9. Viscosity snapshot of an example case (Rh50cc240) in the Sluggish Lid (SL) regime. The model time of the snapshot corresponds to that shown in Fig. 1 for the same SL case. The inset shows a magnified view of the region marked by the dashed-line box, highlighting the V-shaped conjugate plate-scale yielding structure.

Table S1. Reference parameters used in numerical models.

Parameter		Value
Reference viscosity, η_0		1.0×10^{20} Pa s
Surface yield stress (cohesion coefficient), σ_s		Free parameter (See Supplementary Data 1)
Pressure-dependent coefficient, μ		Free parameter (See Supplementary Data 1)
Ideal gas constant, R		8.3145 J/mol/K
Surface temperature		300 K
Bottom temperature		Free parameter (See Supplementary Data 1)
Specific heat capacity		1200 J/kg/K
Internal heating rate		Free parameter (See Supplementary Data 1)
Reference density		3300 kg/m^3
Gravitational acceleration		9.81 m/s^2
Surface thermal expansivity		5×10^{-5} K^{-1}
Surface thermal conductivity		3 W/m/K
Activation energy	upper mantle	Free parameter (See Supplementary Data 1)
	lower mantle	370 kJ/mol
	post-perovskite layer	162 kJ/mol
Activation volume	upper mantle	4.8 cm^3/mol
	lower mantle	3.65 cm^3/mol
	post-perovskite layer	1.4 cm^3/mol
Pressure scale	upper mantle	∞ GPa
	lower mantle	200 GPa
	post-perovskite layer	1610 GPa

Table S2. Phase change parameters for olivine and pyroxene-garnet system with ρ_{surf} the surface density, γ the Clapeyron slope, and $\Delta\rho$ the density jump across any given phase transition.

**Depth (km)**	**Temperature (K)**	**$\Delta\rho$ (kg/m^3)**	**γ (MPa/K)**
Olivine (ρ_{surf} = 3240 kg/m^3)			
410	1600	180	2.5
660	1900	400	−2.5
2740	2300	61.6	10.0
Pyroxene–garnet (ρ_{surf} = 3080 kg/m^3)			
60	1000	350	0
400	1600	150	1.0
720	1900	400	1.0
2740	2300	61.6	10.0

Table S3. Key predictions of high-resolution models (average values and standard deviations of mobility and plateness), and regime classification.

Model	Plateness		Mobility		Base Lid Regime Time Proportion (%)			Tectonic Regime*
	Mean	Standard deviation	Mean	Standard deviation	Mobile Regime	Sluggish/ Squishy Regime	Stagnant Regime	
Rh50cc40_h	0.937	0.037	2.250	0.441	100	0	0	M
Rh200cc300_h	0.193	0.045	0	0	0	0	100	ST
Rh125cc120_h	0.697	0.288	0.537	0.876	21.56	38.76	39.68	E
reference5_h	0.550	0.280	0.204	0.549	8.52	18.46	73.02	ES
reference8_h	0.543	0.294	0.198	0.511	7.04	21.53	71.43	ES
Rh50cc160_h	0.964	0.008	0.405	0.212	2.34	97.66	0	SL
reference1_h	0.918	0.054	2.826	0.389	100	0	0	M
Rh200cc40_h	0.876	0.125	2.036	0.920	87.78	4.40	7.82	M
reference6_h	0.449	0.272	0.199	0.601	7.94	12.70	79.37	ES
Rh50cc200_h	0.982	0.005	0.271	0.180	1.01	98.99	0	SL

*M: Mobile lid; SL: Sluggish lid; E: Episodic lid; ES: Episodic-Squishy lid; ST: Stagnant lid.

Table S4. Key predictions of models with different initial potential temperatures (average values and standard deviations of mobility and plateness), and regime classification.

Model	Plateness		Mobility		Base Lid Regime Time Proportion (%)			Tectonic Regime*
	Mean	Standard deviation	Mean	Standard deviation	Mobile Regime	Sluggish/ Squishy Regime	Stagnant Regime	
Rh50cc40_bt1700	0.909	0.024	2.100	0.409	100	0	0	M
Rh200cc300_bt1700	0.080	0.069	0	0	0	0	100	ST
Rh125cc120_bt1700	0.861	0.170	1.197	1.059	47.58	42.89	9.52	E
reference5_bt1700	0.718	0.270	0.536	0.970	20.36	33.61	46.03	ES
reference8_bt1700	0.390	0.280	0.020	0.072	0	12.70	87.30	PS
Rh50cc160_bt1700	0.951	0.021	0.370	0.346	6.52	93.48	0	SL
reference1_bt1700	0.881	0.070	3.065	0.256	100	0	0	M
Rh200cc40_bt1700	0.821	0.078	2.975	0.402	100	0	0	M
reference6_bt1700	0.704	0.284	0.582	1.035	19.66	29.55	50.79	ES
Rh50cc200_bt1700	0.414	0.304	0.969	0.014	4.30	95.70	0	SL
Rh50cc40_bt2100	0.914	0.050	2.749	0.453	100	0	0	M
Rh200cc300_bt2100	0.071	0.076	0	0	0	0	100	ST
Rh125cc120_bt2100	0.881	0.133	1.568	0.986	67.45	26.20	6.35	E
reference5_bt2100	0.742	0.277	0.735	1.096	23.52	41.56	34.92	ES
reference8_bt2100	0.329	0.241	0.012	0.053	0	7.94	92.06	PS
Rh50cc160_bt2100	0.957	0.024	0.493	0.438	11.51	88.49	0	SL
reference1_bt2100	0.901	0.055	2.969	0.262	100	0	0	M
Rh200cc40_bt2100	0.819	0.077	3.043	0.455	100	0	0	M
reference6_bt2100	0.717	0.273	0.365	0.926	10.47	41.91	47.62	ES
Rh50cc200_bt2100	0.975	0.008	0.289	0.234	2.59	97.41	0	SL

*M: Mobile lid; SL: Sluggish lid; E: Episodic lid; ES: Episodic-Squishy lid; PS: Plutonic-Squishy lid; ST: Stagnant lid.

Reference

1. Karato, S. & Wu, P. Rheology of the Upper Mantle: A Synthesis. *Science* **260**, 771–778 (1993).

2. Karato, S.-I. & Jung, H. Effects of pressure on high-temperature dislocation creep in olivine. *Philos. Mag.* **83**, 401–414 (2003).

3. Christensen, U. R. Heat transport by variable viscosity convection II: pressure influence, non-Newtonian rheology and decaying heat sources. *Phys. Earth Planet. Inter.* **37**, 183–205 (1985).

4. Solomatov, V. S. Scaling of temperature- and stress-dependent viscosity convection. *Phys. Fluids* **7**, 266–274 (1995).

5. Lourenço, D. L., Rozel, A. B., Ballmer, M. D. & Tackley, P. J. Plutonic-Squishy Lid: A New Global Tectonic Regime Generated by Intrusive Magmatism on Earth-Like Planets. *Geochem Geophys Geosystems* **21**, (2020).

6. Bello, L., Coltice, N., Rolf, T. & Tackley, P. J. On the predictability limit of convection models of the Earth's mantle. *Geochem., Geophys., Geosystems* **15**, 2319–2328 (2014).

7. Moresi, L. & Solomatov, V. Mantle convection with a brittle lithosphere: thoughts on the global tectonic styles of the Earth and Venus. *Geophys J Int* **133**, 669–682 (1998).

8. Lourenço, D. L., Rozel, A. & Tackley, P. J. Melting-induced crustal production helps plate tectonics on Earth-like planets. *Earth Planet. Sci. Lett.* **439**, 18–28 (2016).

9. Bercovici, D. & Ricard, Y. Grain-damage hysteresis and plate tectonic states. *Phys. Earth Planet. Inter.* **253**, 31–47 (2016).

10. Weller, M. B. & Lenardic, A. Hysteresis in mantle convection: Plate tectonics systems. *Geophys. Res. Lett.* **39**, (2012).

11. Weller, M. B. & Lenardic, A. On the evolution of terrestrial planets: Bi-stability, stochastic effects, and the non-uniqueness of tectonic states. *Geosci. Front.* **9**, 91–102 (2018).
