# Supplementary material for: Dissecting the puzzle of tectonic lid regimes in terrestrial planets
Source: Nat Commun. 2025 Nov 24;16:10037. doi: 10.1038/s41467-025-65943-1 (PMC12644805; doi:10.1038/s41467-025-65943-1)
Supplement: Supplementary file 2 — Description of Additional Supplementary File [file 41467_2025_65943_MOESM2_ESM.pdf]

Description of Additional Supplementary Files

Supplementary Data 1: Parameters and results of numerical experiments.
